# Effect of procalcitonin-guided antibiotic treatment on clinical outcomes in intensive care unit patients with infection and sepsis patients: a patient-level meta-analysis of randomized trials

**DOI:** 10.1186/s13054-018-2125-7

**Published:** 2018-08-15

**Authors:** Yannick Wirz, Marc A. Meier, Lila Bouadma, Charles E. Luyt, Michel Wolff, Jean Chastre, Florence Tubach, Stefan Schroeder, Vandack Nobre, Djillali Annane, Konrad Reinhart, Pierre Damas, Maarten Nijsten, Arezoo Shajiei, Dylan W. deLange, Rodrigo O. Deliberato, Carolina F. Oliveira, Yahya Shehabi, Jos A. H. van Oers, Albertus Beishuizen, Armand R. J. Girbes, Evelien de Jong, Beat Mueller, Philipp Schuetz

**Affiliations:** 1 Medical University Department, Kantonsspital Aarau, Tellstrasse, CH-5001 Aarau, Switzerland; 2 Faculty of Medicine, University of Basel, Basel, Switzerland; 3 Service de Réanimation Médicale, Université Paris 7-Denis-Diderot, AP-HP, Paris, France; 4 Service de Réanimation Médicale, Université Paris 6-Pierre-et-Marie-Curie, Paris, France; 5 Département d’Epidémiologie Biostatistique et Recherche Clinique, AP-HP, Hôpitaux Universitaires Paris Nord Val de Seine, Paris, France; 6 Department of Anesthesiology and Intensive Care Medicine, Krankenhaus Dueren, Dueren, Germany; 7 Department of Intensive Care, Hospital das Clinicas da Universidade Federal de Minas Gerais, Belo Horizonte, Brazil; 8 Critical Care Department, Hôpital Raymond Poincaré, Assistance Publique - Hôpitaux de Paris, Garches, France; 9 Department of Anesthesiology and Intensive Care Medicine, Jena University Hospital, Jena, Germany; 10 Department of General Intensive Care, University Hospital of Liege, Domaine universitaire de Liège, Liege, Belgium; 11 University Medical Centre, University of Groningen, Groningen, The Netherlands; 12 University Medical Center Utrecht, Utrecht, The Netherlands; 13 Laboratory for Critical Care Research, Critical Care Unit, Hospital Israelita Albert Einstein, São Paulo, Brazil; 14 Department of Internal Medicine, School of Medicine, Universidade Federal de Minas Gerais, Belo Horizonte, Brazil; 15 Critical Care and Peri-operative Medicine, Monash Health, Melbourne, Australia; 16 Faculty of Medicine Nursing and Health Sciences, School of Clinical Sciences, Monash University, Melbourne, Australia; 17 Elisabeth Tweesteden Hospital, Tilburg, The Netherlands; 18 Medisch Spectrum Twente, Enschede, The Netherlands; 19 VU University Medical Centre, Amsterdam, The Netherlands

**Keywords:** Sepsis, Procalcitonin, Antibiotic stewardship, Meta-analysis

## Abstract

**Background:**

The clinical utility of serum procalcitonin levels in guiding antibiotic treatment decisions in patients with sepsis remains unclear. This patient-level meta-analysis based on 11 randomized trials investigates the impact of procalcitonin-guided antibiotic therapy on mortality in intensive care unit (ICU) patients with infection, both overall and stratified according to sepsis definition, severity, and type of infection.

**Methods:**

For this meta-analysis focusing on procalcitonin-guided antibiotic management in critically ill patients with sepsis of any type, in February 2018 we updated the database of a previous individual patient data meta-analysis which was limited to patients with respiratory infections only. We used individual patient data from 11 trials that randomly assigned patients to receive antibiotics based on procalcitonin levels (the “procalcitonin-guided” group) or the current standard of care (the “controls”). The primary endpoint was mortality within 30 days. Secondary endpoints were duration of antibiotic treatment and length of stay.

**Results:**

Mortality in the 2252 procalcitonin-guided patients was significantly lower compared with the 2230 control group patients (21.1% vs 23.7%; adjusted odds ratio 0.89, 95% confidence interval (CI) 0.8 to 0.99; *p* = 0.03). These effects on mortality persisted in a subgroup of patients meeting the sepsis 3 definition and based on the severity of sepsis (assessed on the basis of the Sequential Organ Failure Assessment (SOFA) score, occurrence of septic shock or renal failure, and need for vasopressor or ventilatory support) and on the type of infection (respiratory, urinary tract, abdominal, skin, or central nervous system), with interaction for each analysis being > 0.05. Procalcitonin guidance also facilitated earlier discontinuation of antibiotics, with a reduction in treatment duration (9.3 vs 10.4 days; adjusted coefficient −1.19 days, 95% CI −1.73 to −0.66; *p* <  0.001).

**Conclusion:**

Procalcitonin-guided antibiotic treatment in ICU patients with infection and sepsis patients results in improved survival and lower antibiotic treatment duration.

**Electronic supplementary material:**

The online version of this article (10.1186/s13054-018-2125-7) contains supplementary material, which is available to authorized users.

## Background

Sepsis, defined as life-threatening organ dysfunction caused by a dysregulated host response to infection, remains a major healthcare problem worldwide and affects millions of people each year [1, 2]. Early identification and appropriate initial management including the start of antibiotic treatment and fluid resuscitation improves outcomes [2, 3]. In addition, monitoring of patients during treatment both for timely escalation of therapy in case of treatment failure and de-escalation in case of a favorable treatment response has an important impact on patient recovery. [1, 2] This also includes early de-escalation or cessation of antibiotic treatment once a patient’s condition has stabilized, with signs indicating progression towards the resolution of infection.

Given that clinical signs for monitoring patients with sepsis can be ambiguous, the use of additional biomarkers mirroring specific physiopathological pathways has been proposed. In this context, serum procalcitonin (PCT) has emerged as a sensitive biomarker that provides prognostic information in patients with infections, and thus may improve sepsis management [4, 5]. Multiple studies have demonstrated that PCT levels increase in response to bacterial infection and decrease during recovery [4, 5]. Reflecting the host response to a bacterial infection, PCT thus provides important adjunctive information in addition to traditional clinical and diagnostic parameters [6].

Multiple trials have investigated the benefits of using serum PCT levels to guide whether and for how long antibiotic therapy is used—a process referred to as PCT-guided antibiotic stewardship—in patients with infection in the intensive care unit (ICU) [1, 7–17]. While most trials have focused on the effects of PCT guidance on antibiotic usage, a recent large trial from the Netherlands reported lower mortality following PCT-guided therapy [10]. However, conclusive evidence on the safety of this approach across different types of infection and sepsis severities has been limited due to largely insufficient statistical power in most of the individual trials. Moreover, meta-analyses focusing on the impact of PCT-guided antibiotic stewardship in sepsis patients on clinical outcomes overall and within patient subgroups based on infection type and severity have been inconclusive [18, 19]. A noteworthy limitation of these meta-analyses was that they were based on aggregate data, limiting opportunities to harmonize outcome definitions among trials and to investigate the impact of PCT guidance in different patient subgroups.

To address this significant drawback of earlier meta-analyses, we performed a meta-analysis of individual patient data from 11 randomized-controlled trials (RCTs) to assess the safety of using PCT to guide antibiotic decisions in ICU patients with infection and different sepsis severities and with the involvement of different organs.

## Methods

### Definition of patient population and trial selection

For this meta-analysis focusing on PCT-guided antibiotic management in critically ill patients with sepsis, we updated the database of a previous individual patient data meta-analysis which was limited to patients with respiratory infections only [20, 21]. Trial selection and data collection were performed following the initial protocol published in the Cochrane Library [22] and the report was prepared according to PRISMA individual participant data (IPD) guidelines [23, 24].

For this analysis, we included all patients residing in an ICU with any type of systemic infection. We thus excluded patients not treated in the ICU and also one trial that focused on ventilator-associated pneumonia because these patients may not have systemic bacterial infections [25]. Furthermore, pediatric trials and those not using PCT to guide initiation and duration of antibiotic treatment were excluded from this analysis.

Given that the definition of sepsis has changed over time [26], and is used differently among researchers and clinicians, we decided to focus primarily on trials involving patients presenting with infections to the critical care unit. However, we performed a subgroup analysis on patients meeting the sepsis 3 definition [26]. We also stratified the analysis based on sepsis severity and type of organ involved. Individual patient data were collected from eligible RCTs which assessed adults meeting these criteria.

### Trial search and data collection

The search strategy for this review was updated in February 2018 in collaboration with personnel from the Cochrane collaboration and executed in all databases from the date of their inception to February 2018. All references were also screened for eligibility. Databases searched included the Cochrane Central Register of Controlled Trials (CENTRAL; January 2017, issue 1), MEDLINE Ovid (1966 to February 2017), and Embase.com (1980 to February 2017). We applied no language or publication restrictions.

Two authors (YW and MAM) independently assessed trial eligibility based on titles, abstracts, and full-text reports, with further information being obtained directly from investigators as needed. Study protocols, case report forms, and unedited databases containing individual patient data were requested from investigators of all eligible trials. Data from each trial were first checked against reported results and queries were resolved with the principal investigator, trial data manager, or statistician. Data were assessed in a consistent manner across all trials, with standard definitions and parameters, and thus mortality rates differed slightly from previous reports. In accordance with the Cochrane methodology, we used the Grading of Recommendations, Assessment, Development, and Evaluation (GRADE) approach to assess risk of selection bias, performance bias, detection bias, attrition bias, reporting bias, and other types of bias [27]. The grading was performed by two authors (YW and MAM) and, in case of conflicting results, grading was discussed with another author (PS) and within the meta-analysis group.

### Patients and endpoints

All patients with a suspected or proven infection as the main diagnosis treated in the ICU who were included in a previous trial and randomized either to PCT-guided care or a control group were included in the overall analysis. The primary safety endpoint was all-cause mortality within 30 days of randomization. For trials with a shorter follow-up period, the available information was used (e.g., mortality at the time of hospital discharge). The main efficacy endpoint was antibiotic treatment duration. Other clinical endpoints included length of hospital stay and length of ICU stay.

### Statistical analysis

The analysis follows a study protocol similar to a previous protocol published in the Cochrane Library [22] but we specified the main analysis and subgroup analysis for the sepsis patient population. For the primary endpoint (mortality), odds ratios (ORs) and 95% confidence intervals (CIs) were calculated using multivariable hierarchical logistic regression [28, 29]. Variables in the multivariate analysis included treatment arm, age, gender, and type of infection. To control for within- and between-trial variability, a “trial” variable was added to the model as a random effect. Corresponding linear and logistic regression models were fitted for continuous and binary secondary endpoints, respectively. Analyses followed the intention-to-treat principle by analyzing patients in groups to which they were randomized. Censoring was used for patients with a follow-up < 30 days for the time-to-event analyses.

We performed a prespecified subgroup analysis on patients meeting the sepsis 3 definition, i.e., defining sepsis as life-threatening organ dysfunction due to a dysregulated host response to infection, and, as such, requires at least one organ dysfunction (i.e., at least one organ with a Sequential Organ Failure Assessment (SOFA) score above or equal to 2 [26]). Additional prespecified subgroup analyses were performed for sepsis severity (septic shock), treatment modality in the ICU, and type of organ infected. We tested for subgroup effects by adding interaction terms to the model. All statistical analyses were performed using Stata version 9.2 (College Station, Texas, USA) and Review Manager version 5.3.

## Results

### Results of the search and characteristics of included trials

**Fig. 1:**
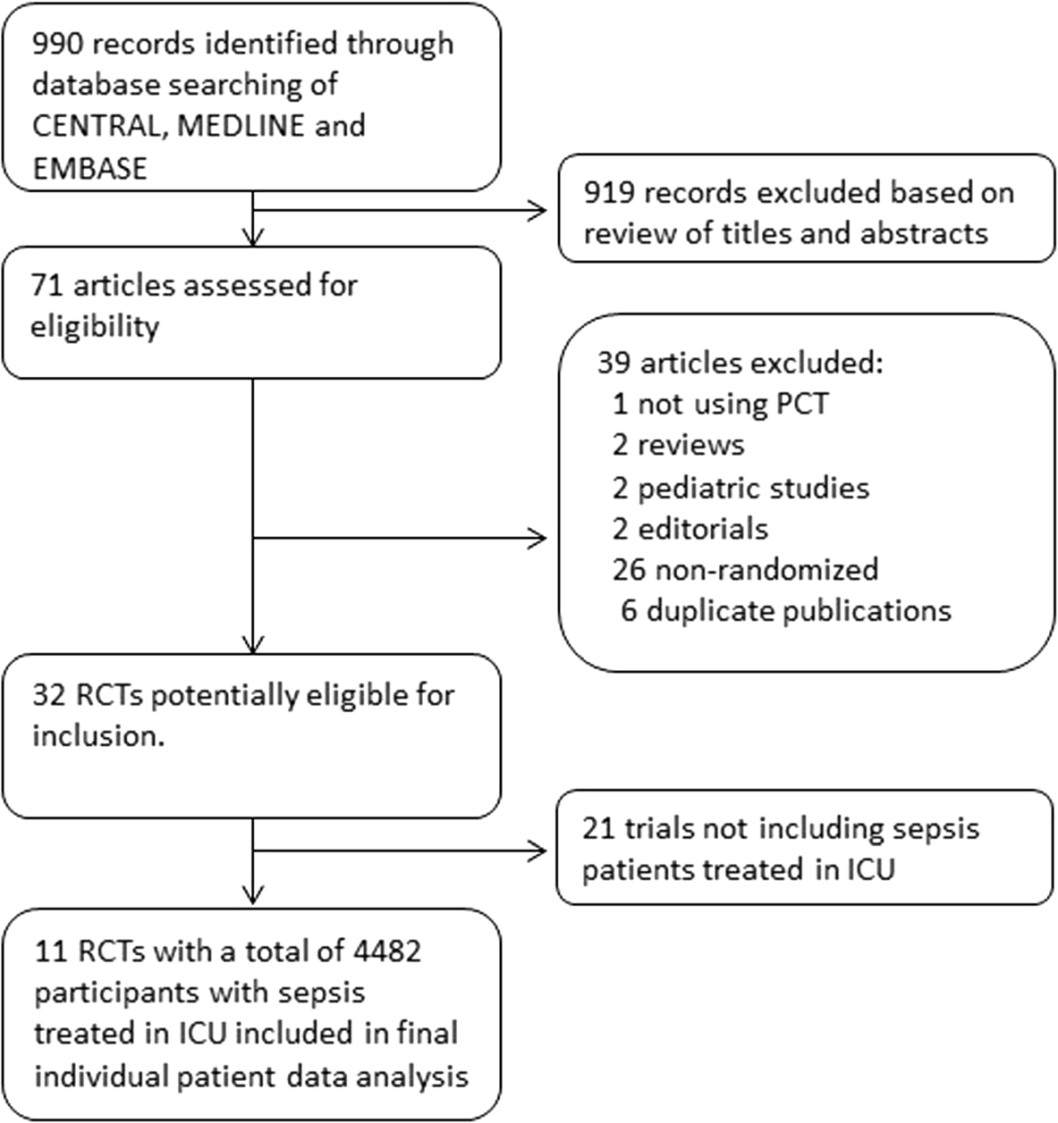
Study flow diagram. ICU intensive care unit, PCT procalcitonin, RCT randomized controlled trial

Data from 4482 individual patients enrolled in 11 of 32 potentially eligible trials were obtained and included in the meta-analysis (Fig. 1). A total of 21 trials whose patients did not have sepsis treated in the ICU and which thus did not meet our inclusion criteria were excluded. Included trials were conducted in seven countries including Switzerland, Germany, France, the Netherlands, Brazil, Belgium, and Australia (Table 1). There were two trials from surgical ICUs with postoperative sepsis patients, while the remaining nine trials focused on medical or mixed ICU patients with sepsis. All trials focused on sepsis patients but, as demonstrated in Table 1, definition of patients varied somewhat. The largest trials were those by De Jong (*n* = 1546) [10], Bloos (*n* = 1089) [8], and Bouadma (*n* = 621) [9]. The PCT algorithms used in the different trials were similar and mainly focused on early discontinuation of antibiotics if PCT dropped below 0.5 μg/L or by 80% from the peak level.

**Table 1:** Characteristics of included trials

First author (year)	Country	Setting, type of trial	Patients included	Follow-up	Clinical diagnosis	Type of procalcitonin algorithm; procalcitonin cutoffs used (μg/L)^a^	Compliance with the PCT protocol
Annane (2013) [7]	France	ICU, multicenter	62	Hospital stay	Severe sepsis without overt source of infection and negative blood culture	Initiation and duration; R against AB: < 0.5 (< 0.25); R for AB: > 0.5 (> 5.0)	63% adherence
Bloos (2016) [8]	Germany	ICU, multicenter	1089	3 months	Severe sepsis or septic shock (SIRS and documented infection + criteria for severe sepsis/septic shock)	Discontinuation at days 4, 7, and 10; R against AB: < 1.0 or > 50% drop over previous value	49.6% adherence
Bouadma (2010) [9]	France	ICU, multicenter	621	2 months	Critically ill patients with assumed/proven bacterial infection	Initiation and duration; R against AB: < 0.5 (< 0.25); R for AB: > 0.5 (> 1.0)	47% adherence
De Jong (2016) [10]	The Netherlands	ICU, multicenter	1546	1 year	Critically ill patients with assumed infection	Duration; R against AB: < 0.5 or > 80% drop over peak value	44% adherence
Deliberato (2013) [11]	Brazil	ICU, single center	81	ICU discharge or 14 days postrandomization	Sepsis patients with microbiologically confirmed bacterial infection	Duration; R against AB: < 0.5 or > 90% drop over peak value	47.6% adherence
Hochreiter (2009) [14]	Germany	Surgical ICU, single center	110	Hospital stay	Sepsis (SIRS and documented infection)	Duration; R against AB: < 1 or > 65% drop over 3 days	not reported
Layios (2012) [15]	Belgium	ICU, single center	379	1 month	Critically ill patients with assumed infection	Initiation; R against AB: < 0.5 (< 0.25); R for AB: > 0.5 (> 1.0)	46.3% adherence
Nobre (2008) [17]	Switzerland	ICU, single center	79	1 month	Severe sepsis or septic shock	Duration; R against AB: < 0.5 (< 0.25) or > 80% drop over peak value; R for AB: > 0.5 (> 1.0)	81% adherence
Oliveira (2013) [16]	Brazil	ICU, multicenter	94	28 days or hospital discharge	Severe sepsis or septic shock (SOFA score > 10 and/or bacteremia)	Discontinuation; Initial < 1.0: R against AB: 0.1 at day 4; Initial > 1.0: R against: > 90% drop over peak value	87.8% adherence
Schroeder (2009) [13]	Germany	Surgical ICU, single center	27	Hospital stay	Severe sepsis following abdominal surgery (SIRS and documented infection + criteria for severe sepsis/septic shock)	Duration; R against AB: < 1 or > 65% drop over 3 days	not reported
Shehabi (2014) [1]	Australia	ICU, multicenter	394	3 months	Sepsis (SIRS and documented infection)	Duration; R against AB: < 0.25 (< 0.1) or > 90% drop over peak value	97% adherence

*AB* antibiotic, *ICU* intensive care unit, *PCT* procalcitonin, *R* recommendation, *SIRS* systemic inflammation response system, *SOFA* Sequential Organ Failure Assessment

^a^ Cutoffs are listed as recommendation (strong recommendation)

The quality of trials according to GRADE criteria was moderate to high (see Additional file 1). There was a low risk of selection bias, attrition bias, and reporting bias. Trials did not blind caregivers and patients with regard to the intervention and only about one-third of the trials featured blinded outcome assessment. There was no evidence of publication bias based on inspection of the funnel plot (see Additional file 1). We also found variable adherence to the PCT protocols ranging from 44% to 97% (Table 1).

Baseline characteristics of individual patients were similar between the PCT and control groups. About 50% of patients had sepsis due to infection of the lung, followed by abdominal infection (18%) and urinary tract infection (5%). The mean SOFA score was 7.4 points and more than two-thirds of patients were on vasopressors and/or ventilation support. Table 2 lists additional baseline characteristics stratified according to randomization. There were no statistical differences between the two groups.

**Table 2:** Baseline characteristics of included patients

Parameter	Control group (*n* = 2230)	PCT group (*n* = 2252)
Demographics		
Age (years)	64.1 ± 15.0	63.5 ± 15.2
Male gender	1281 (57.5%)	1273 (56.5%)
Primary focus of infection		
Respiratory	1101 (49.4%)	1102 (48.9%)
Urinary	129 (5.8%)	118 (5.2%)
Abdominal	417 (18.7%)	391 (17.4%)
Skin/soft tissue	41 (1.8%)	32 (1.4%)
CNS	35 (1.6%)	38 (1.7%)
Other/unknown	440 (19.7%)	519 (23.0%)
Genital/gynecologic	8 (0.4%)	3 (0.1%)
Catheter-related	14 (0.6%)	16 (0.7%)
Bloodstream	36 (1.6%)	25 (1.1%)
Upper respiratory	9 (0.4%)	8 (0.4%)
Vital signs		
Temperature (°C)	37.7 ± 1.2	37.8 ± 1.1
Sepsis score		
Meeting sepsis 3 definition	1630 (73.1%)	1605 (71.3%)
SOFA score (points)	7.4 ± 4.0	7.3 ± 4.1
Additional sepsis support		
Vasopressor use	1593 (76.3%)	1606 (76.7%)
Ventilation support	1434 (68.1%)	1478 (69.4%)
Renal replacement	767 (34.4%)	757 (33.6%)

Values are presented as mean ± standard deviation or *n* (%) as appropriate

*CNS* central nervous system, *PCT* procalcitonin, *SD* standard deviation, *SOFA* Sequential Organ Failure Assessment

### Primary safety endpoint: mortality

**Fig. 2:**
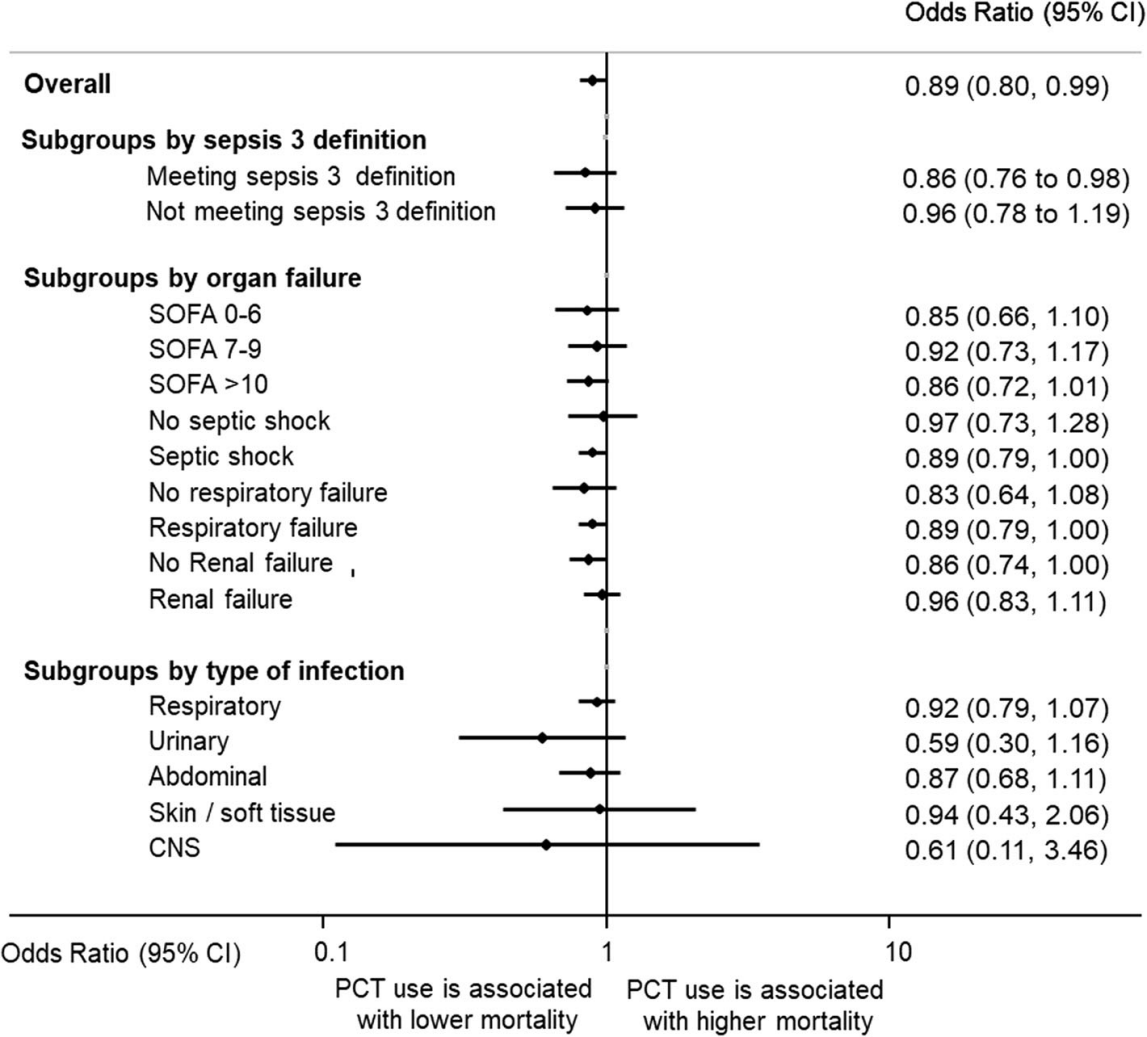
Forest plot showing 30-day mortality. Association of procalcitonin (PCT)-guided antibiotic stewardship and mortality in predefined subgroups. CI confidence interval, CNS central nervous system, SOFA Sequential Organ Failure Assessment

There were 529 deaths among 2230 control group patients (23.7%) compared with 475 deaths among 2252 PCT-guided patients (21.1%), resulting in significantly lower mortality in the PCT group (adjusted OR 0.89, 95% CI 0.80 to 0.99; *p* = 0.03) (Fig. 2 and Table 3). This effect was consistent in patients meeting the sepsis 3 definition, and across different severity groups based on SOFA score, presence or absence of septic shock, ventilatory failure, and renal support (*p* > 0.05 for each interaction, indicating no significant difference due to subgroup effect). The effects on mortality were also consistent among different types of infections including respiratory tract, urinary, abdominal, skin/soft tissue, and central nervous system (CNS) infections (*p* > 0.05 for each interaction) (Table 4).

**Table 3:** Clinical endpoints overall and stratified by sepsis severity and use of sepsis support

	Control group	PCT group	Adjusted OR or difference (95% CI)^a^, *p* value	*p* value for interaction
	2230	2252		
Overall				
Antibiotic therapy (days)	10.4 ± 9.7	9.3 ± 9.2	−1.19 (−1.73 to −0.66), *p* < 0.001	
30-day mortality	529 (23.7%)	475 (21.1%)	0.89 (0.8 to 0.99), *p* = 0.03	
Length of hospital stay (days)	28.7 ± 27.9	28.6 ± 27.9	0.09 (−1.51 to 1.7), *p* = 0.908	
Length of ICU stay (days)	14.7 ± 16.3	14.8 ± 16.5	0.04 (−0.9 to 0.99), *p* = 0.928	
Subgroup by sepsis definition				
Meeting sepsis 3 definition	1630	1605		
Antibiotic therapy (days)	10.5 ± 9.2	9.3 ± 8.9	−1.22 (−1.82 to −0.62), *p* < 0.001	0.915
30-day mortality	397 (24.4%)	338 (21.1%)	0.86 (0.76 to 0.98), *p* = 0.022	0.388
Length of hospital stay (days)	29.5 ± 27.9	29.6 ± 29.4	0.07 (−1.89 to 2.03), *p* = 0.946	0.891
Length of ICU stay (days)	14.1 ± 15.5	14.5 ± 17.0	0.37 (−0.74 to 1.48), *p* = 0.51	0.246
Not meeting sepsis 3 definition	600	647		
Antibiotic therapy (days)	10.2 ± 10.9	9.1 ± 9.9	−1.13 (−2.27 to 0.01), *p* = 0.052	0.915
30-day mortality	132 (22.0%)	137 (21.2%)	0.96 (0.78 to 1.19), *p* = 0.717	0.388
Length of hospital stay (days)	26.4 ± 27.9	26.2 ± 23.7	0.16 (−2.53 to 2.84), *p* = 0.909	0.891
Length of ICU stay (days)	16.5 ± 18.3	15.6 ± 15.2	−0.82 (−2.65 to 1.01), *p* = 0.378	0.246
Subgroup by organ failure				
SOFA 0 to 6	763	776		
Antibiotic therapy (days)	10.9 ± 10.3	8.1 ± 8.2	−2.62 (−3.51 to −1.73), *p* < 0.001	< 0.001
30-day mortality	105 (13.8%)	91 (11.7%)	0.85 (0.66 to 1.1), *p* = 0.227	0.991
Length of hospital stay (days)	28.6 ± 28.6	26.8 ± 25.8	−1.96 (−4.65 to 0.72), *p* = 0.152	0.138
Length of ICU stay (days)	12.9 ± 16.7	12.1 ± 15.6	−0.92 (−2.52 to 0.69), *p* = 0.263	0.083
SOFA 7 to 9	474	445		
Antibiotic therapy (days)	10.1 ± 8.2	10.2 ± 9.4	0.01 (−1.1 to 1.11), *p* = 0.988	0.003
30-day mortality	112 (23.6%)	96 (21.6%)	0.92 (0.73 to 1.17), *p* = 0.512	0.601
Length of hospital stay (days)	30.7 ± 26.4	29.4 ± 25.9	−1.14 (−4.52 to 2.24), *p* = 0.508	0.543
Length of ICU stay (days)	14.0 ± 14.3	14.1 ± 15.3	0.23 (−1.66 to 2.12), *p* = 0.813	0.862
SOFA 10 to 24	486	486		
Antibiotic therapy (days)	10.7 ± 9.1	10.0 ± 8.8	−0.63 (−1.71 to 0.45), *p* = 0.256	0.125
30-day mortality	190 (39.1%)	161 (33.1%)	0.86 (0.72 to 1.01), *p* = 0.065	0.576
Length of hospital stay (days)	29.4 ± 27.5	32.6 ± 35.5	3.21 (−0.76 to 7.18), *p* = 0.113	0.024
Length of ICU stay (days)	15.6 ± 15.4	17.7 ± 19.2	2.08 (−0.08 to 4.24), *p* = 0.059	0.036
No septic shock (no vasopressor use)	495	489		
Antibiotic therapy (days)	11.2 ± 9.9	9.8 ± 9.2	−1.35 (−2.53 to −0.17), *p* = 0.025	0.586
30-day mortality	79 (16.0%)	76 (15.5%)	0.97 (0.73 to 1.28), *p* = 0.823	0.512
Length of hospital stay (days)	24.9 ± 26.3	23.2 ± 20.6	−1.49 (−4.35 to 1.38), *p* = 0.309	0.258
Length of ICU stay (days)	13.2 ± 15.6	12.7 ± 14.2	−0.43 (−2.25 to 1.39), *p* = 0.642	0.47
Septic shock (with vasopressor use)	1593	1606		
Antibiotic therapy (days)	10.4 ± 9.8	9.3 ± 9.4	−1.03 (−1.68 to −0.39), *p* = 0.002	0.586
30-day mortality	420 (26.4%)	376 (23.4%)	0.89 (0.79 to 1), *p* = 0.051	0.512
Length of hospital stay (days)	30.1 ± 27.0	30.6 ± 26.3	0.6 (−1.23 to 2.43), *p* = 0.52	0.258
Length of ICU stay (days)	15.0 ± 16.1	15.4 ± 17.0	0.34 (−0.8 to 1.49), *p* = 0.557	0.47
No respiratory failure (invasive ventilation support)	672	651		
Antibiotic therapy (days)	11.0 ± 11.4	9.5 ± 9.6	−1.43 (−2.49 to − 0.37), *p* = 0.008	0.467
30-day mortality	104 (15.5%)	83 (12.7%)	0.83 (0.64 to 1.08), *p* = 0.158	0.633
Length of hospital stay (days)	25.4 ± 21.7	25.3 ± 23.3	−0.01 (−2.33 to 2.31), *p* = 0.993	0.788
Length of ICU stay (days)	11.7 ± 13.5	12.4 ± 14.2	0.53 (− 0.86 to 1.92), *p* = 0.456	0.433
Respiratory failure (invasive ventilation support)	1434	1478		
Antibiotic therapy (days)	10.2 ± 9.0	9.2 ± 9.2	−1.09 (−1.73 to −0.45), *p* = 0.001	0.467
30-day mortality	401 (28.0%)	373 (25.2%)	0.89 (0.79 to 1), *p* = 0.058	0.633
Length of hospital stay (days)	30.9 ± 30.9	30.3 ± 26.0	−0.39 (−2.42 to 1.65), *p* = 0.708	0.788
Length of ICU stay (days)	16.2 ± 17.5	16.0 ± 17.5	−0.3 (−1.56 to 0.95), *p* = 0.637	0.433
No renal replacement therapy	1463	1495		
Antibiotic therapy (days)	9.4 ± 9.2	8.4 ± 8.6	−1.02 (−1.64 to −0.39), p = 0.001	0.475
30-day mortality	301 (20.6%)	265 (17.7%)	0.86 (0.74 to 1), *p* = 0.046	0.553
Length of hospital stay (days)	29.8 ± 31.1	28.4 ± 25.5	−1.41 (−3.4 to 0.59), *p* = 0.168	0.026
Length of ICU stay (days)	14.6 ± 17.4	14.1 ± 15.9	−0.64 (−1.82 to 0.53), *p* = 0.284	0.077
Renal replacement therapy	767	757		
Antibiotic therapy (days)	12.3 ± 10.2	10.9 ± 10.0	−1.45 (−2.44 to −0.46), *p* = 0.004	0.475
30-day mortality	228 (29.7%)	210 (27.7%)	0.96 (0.83 to 1.11), *p* = 0.584	0.553
Length of hospital stay (days)	26.6 ± 20.5	29.1 ± 32.2	2.97 (0.31 to 5.63), *p* = 0.028	0.026
Length of ICU stay (days)	14.9 ± 14.0	16.2 ± 17.5	1.43 (−0.16 to 3.02), *p* = 0.079	0.077

Values are presented as mean ± standard deviation or *n* (%) as appropriate

*CI* confidence interval, *ICU* intensive care unit, *OR* odds ratio, *PCT* procalcitonin, *SOFA* Sequential Organ Failure Assessment

^a^ Multivariable hierarchical regression with outcome of interest as dependent variable and trial as a random effect

**Table 4:** Clinical endpoints stratified by type of infection

	Control group	PCT group	Adjusted OR or difference (95% CI)^a^, *p* value	*p* value for interaction
Subgroup by type of infection (suspected infection site)				
Respiratory	1101	1102		
Antibiotic therapy (days)	9.9 ± 7.8	8.5 ± 7.8	−1.36 (−1.99 to −0.73), *p* < 0.001	0.582
30-day mortality	262 (23.8%)	243 (22.1%)	0.92 (0.79 to 1.07), *p* = 0.299	0.466
Length of hospital stay (days)	28.2 ± 27.7	27.7 ± 24.7	−0.21 (−2.36 to 1.94), *p* = 0.849	0.668
Length of ICU stay (days)	15.1 ± 16.6	15.3 ± 17.5	0.19 (−1.24 to 1.61), *p* = 0.798	0.858
Urinary	129	118		
Antibiotic therapy (days)	12.5 ± 12.4	11.0 ± 12.2	−1.62 (−4.6 to 1.36), *p* = 0.286	0.786
30-day mortality	21 (16.3%)	11 (9.3%)	0.59 (0.3 to 1.16), *p* = 0.128	0.215
Length of hospital stay (days)	29.5 ± 25.4	25.1 ± 21.7	−4.08 (−9.7 to 1.54), *p* = 0.154	0.209
Length of ICU stay (days)	14.3 ± 20.5	11.2 ± 13.6	−2.49 (−6.68 to 1.7), *p* = 0.244	0.123
Abdominal	417	391		
Antibiotic therapy (days)	10.5 ± 10.6	11.0 ± 11.9	0.55 (−0.96 to 2.06), *p* = 0.477	0.005
30-day mortality	109 (26.1%)	89 (22.8%)	0.87 (0.68 to 1.11), *p* = 0.266	0.821
Length of hospital stay (days)	30.5 ± 27.7	32.1 ± 27.8	1.62 (−2.18 to 5.41), *p* = 0.404	0.361
Length of ICU stay (days)	15.1 ± 15.3	15.7 ± 16.4	0.42 (−1.76 to 2.6), *p* = 0.704	0.634
Skin/soft tissue	41	32		
Antibiotic therapy (days)	12.9 ± 15.8	8.6 ± 8.5	−4.57 (−10.36 to 1.23), p = 0.122	0.159
30-day mortality	11 (27%)	8 (25%)	0.94 (0.43 to 2.06), *p* = 0.874	0.918
Length of hospital stay (days)	26.0 ± 25.8	20.9 ± 23.4	−4.17 (−15.4 to 7.06), *p* = 0.467	0.442
Length of ICU stay (days)	10.4 ± 9.4	10.9 ± 12.7	0.83 (−4.18 to 5.84), *p* = 0.747	0.916
Central nervous system	35	38		
Antibiotic therapy (days)	11.7 ± 7.7	10.4 ± 7.7	−1.7 (−5.04 to 1.63), *p* = 0.317	0.958
30-day mortality	3 (9%)	2 (5%)	0.61 (0.11 to 3.46), *p* = 0.58	0.692
Length of hospital stay (days)	31.3 ± 22.8	30.8 ± 25.7	−0.44 (−11.48 to 10.6), *p* = 0.938	0.954
Length of ICU stay (days)	10.8 ± 14.0	13.7 ± 13.1	2.91 (−3.22 to 9.04), *p* = 0.352	0.457

Values are presented as mean ± standard deviation or *n* (%) as appropriate

*CI* confidence interval, *ICU* intensive care unit, *OR* odds ratio, *PCT* procalcitonin

^a^Multivariable hierarchical regression with outcome of interest as dependent variable and trial as a random effect

As an additional sensitivity analysis, a meta-analysis of the aggregate results of all trials was conducted (see Additional file 1). The point estimate for mortality in this analysis was similar to that seen in the individual patient data analysis but did not reach statistical significance (OR 0.88, 95% CI 0.76 to 1.01). Heterogeneity was low, suggesting robust effects (*I*^2^ = 0%).

As an additional sensitivity analysis, we restricted the analysis to trials reporting ≥ 28 days mortality, thereby excluding three trials where this information was not assessed (Table 1). Again, results were similar to the main results.

### Primary efficacy endpoint: antibiotic use

A moderate reduction in total antibiotic treatment duration resulted from PCT guidance (mean 10.4 ± 9.7 vs 9.3 ± 9.2 days; adjusted regression coefficient −1.19 days, 95% CI −1.73 to −0.66; *p* < 0.001) (Table 3). Effects were similar in patients meeting the sepsis 3 definition. There was some evidence of effect modification with stronger effects in patients with lower sepsis severity based on SOFA score (−2.62 days in patients with SOFA score of 0–6 points vs 0.01 days in patients with SOFA score of 7–9 and −0.63 days in patients with SOFA scores of 10–24 (*p* for the interaction < 0.05). Similarly, in patients with abdominal infection, PCT-guided therapy did not reduce duration of treatment compared with patients with all other infected organs, with the strongest effects being seen in patients with skin and soft tissue infections (−4.6 days, 95% CI −10.36 to 1.23; *p* = 0.122).

### Length of stay

Length of hospital stay (adjusted regression coefficient 0.09 days, 95% CI −1.51 to 1.7; *p* = 0.908) and ICU stay (adjusted regression coefficient 0.04 days, 95% CI −0.9 to 0.99; *p* = 0.928) were similar in the PCT and control groups overall and in most subgroup analyses. Patients in the highest SOFA categories had longer lengths of ICU and hospital stay if PCT guidance had been used (3.21 days, 95% CI −0.76 to 7.18, and 2.08 days, 95% CI −0.08 to 4.24, respectively). In addition, patients with renal failure (renal support) had longer lengths of hospital stay while patients with no renal failure had shorter stays (2.97 days vs −1.41 days).

## Discussion

This meta-analysis of individual patient data from 11 randomized trials that included 4482 patients with infection treated in ICUs revealed lower mortality associated with PCT-guided therapy, confirming the results of a large Dutch trial [10]. This effect was consistent in sepsis patients based on the sepsis 3 definition, and across different severities and types of sepsis based on whether PCT-guided treatment was employed, and also across different types of infection. Moreover, PCT guidance was also associated with a modest reduction in exposure to antibiotics through shorter treatment durations and earlier discontinuation. However, PCT guidance did not have an effect on length of ICU or hospital stay.

Early diagnosis combined with initiation of appropriate antibiotic treatment remains the cornerstone of sepsis care [30]. Once treatment is initiated, close monitoring of patients is of the utmost importance to identify patients with a favorable disease course who are at low risk for complications so that one may consider early cessation of antibiotic therapy. Daily assessment of patient risk using objective prognostic parameters is therefore important. In addition to clinical parameters, blood markers may aid in patient monitoring [31–34]. While serum lactate is a commonly used biomarker that may help guide resuscitation measures [35], PCT has been previously demonstrated to be helpful in assessing the response to antimicrobial treatment and in aiding antibiotic stewardship decisions [36–38]. Based on multiple randomized trials integrated in an aggregate patient data meta-analysis, the US Food and Drug Administration (FDA) has recently approved the PCT assay for the purpose of guiding antibiotic therapy in the context of sepsis [18, 39]. However, since sepsis is not a well-defined disease but a heterogenous syndrome arising from different possible organs being infected and with different clinical presentations based on severity, the question of safety and efficacy of PCT guidance in patients with sepsis overall, and in subgroups based on severity and organs involved, is relevant. In this context, our finding that lower mortality and shorter antibiotic courses are associated with PCT-guided care is reassuring.

We have recently published a similar individual patient data meta-analysis looking at patients with different types and severities of respiratory infections [20, 40]. Similar to what was seen in the current analysis, patients with respiratory infections also benefited from PCT-guided therapy, experiencing lower mortality and needing significantly reduced antibiotic exposure. The current analysis further expands these findings, also showing similar effects in subgroups of other types of infections—an analysis that was not possible in all previous meta-analyses based on aggregate data. While the effects seem similar between different subgroups, we did not find PCT to be associated with reduced antibiotic use in patients with abdominal infections and those with renal impairment. It is possible that, in these two patient groups, the PCT kinetics are impaired by bacterial translocation due to mucositis even if the initial infection has resolved, and by the slower decline of PCT in patients with renal impairment. Additional investigations will be needed to understand whether PCT algorithms require further adaptation in these two patient groups.

Although we have no definitive explanations for the positive effects of PCT-guided antibiotic treatment on mortality, several mechanisms seem plausible. First, PCT provides additional prognostic information in patients with sepsis and may influence site-of-care decisions and timing of discharge [17]. The MOSES study investigating PCT kinetics over 72 h in several US emergency departments validated the prognostic potential of PCT independent of other prognostic scores [4]. Hence, early identification of treatment nonresponders has the potential to prevent adverse events, although this was not true in the PASS trial, a study investigating whether escalation of diagnostic and therapeutic measures based on high PCT levels would decrease mortality [41]. Second, prolonged antibiotic exposure has a toxic effect and thus may increase risk of treatment failure in control patients [42, 43]. Unexpected PCT results may also prompt physicians to further examine patients and look for additional explanations and illnesses. Finally, several observational studies have reported lower mortality and risk of treatment failure associated with early antibiotic de-escalation in patients with sepsis, although pathophysiological mechanisms are incompletely understood [37, 44].

This analysis is based on a comprehensive literature search and retrieval of all relevant trials and a network that permitted inclusion of individual patient data from most eligible trials. We also standardized definitions across trials and were able to perform appropriate subgroup analyses looking at different sepsis severities and types of infection. Limitations of this analysis include incomplete adherence to the PCT algorithm among the included trials, exclusion of immunocompromised patients in most trials, and heterogeneity among trials with regard to patient populations and follow-up of patients. In addition, cost-effectiveness analyses need to be undertaken to determine whether using PCT-guided care is a cost-effective intervention [45].

## Conclusion

In conclusion, PCT-guided antibiotic treatment in ICU patients with infection results in improved survival and shorter antibiotic treatment duration. Effects were similar in sepsis patients and among subgroups based on sepsis severity, sepsis treatment modalities, and type of infection. Whether the reduction in antibiotic exposure fully explains the mortality effects seen in our data needs to be investigated in future trials. These findings have substantial clinical and public health implications.

## Additional file

Additional file 1: **Figure S1.** Assessment of risk of bias in included trials. **Figure S2.** Forrest plot based on aggregate data. **Figure S3.** Funnel plots regarding possible publication bias. (PDF 57 kb)
