# Supplementary material for: Effect of procalcitonin-guided antibiotic treatment on clinical outcomes in intensive care unit patients with infection and sepsis patients: a patient-level meta-analysis of randomized trials
Source: Crit Care. 2018 Aug 15;22:191. doi: 10.1186/s13054-018-2125-7 (PMC6092799; doi:10.1186/s13054-018-2125-7)

F1. Assessment of risk of bias in included trials

	Random sequence generation (selection bias)	Allocation concealment (selection bias)	Blinding of participants and personnel (performance bias)	Blinding of outcome assessment (detection bias)	Incomplete outcome data (attrition bias)	Selective reporting (reporting bias)	Other bias
Annane 2013	+	+	?	−	+	+	?
Bloos 2016	+	+	?	−	+	+	?
Bouadma 2010	+	+	?	+	+	+	?
De Jong 2016	+	+	?	−	+	+	−
Deliberato 2013	+	+	?	−	+	+	−
Hochreiter 2009	−	−	?	−	+	+	?
Layios 2012	?	?	?	+	+	?	−
Nobre 2008	+	+	?	−	+	+	+
Oliveira 2013	+	+	?	−	+	+	+
Schroeder 2009	?	−	?	−	+	+	?
Shehabi 2014	+	+	?	+	+	+	+

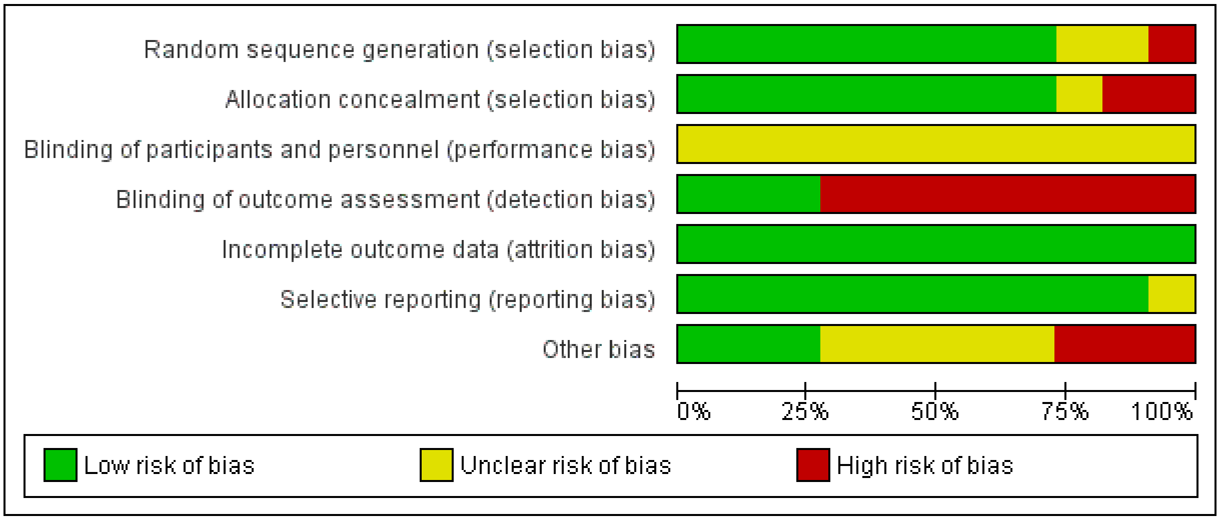
Random sequence generation (selection bias)
Allocation concealment (selection bias)
Blinding of participants and personnel (performance bias)
Blinding of outcome assessment (detection bias)
Incomplete outcome data (attrition bias)
Selective reporting (reporting bias)
Other bias
0%
25%
50%
75%
100%
Low risk of bias
Unclear risk of bias
High risk of bias

F2 Forrest plot based on aggregate data

Study or Subgroup	PCT-guided care Events	PCT-guided care Total	Controls Events	Controls Total	Weight	Odds Ratio M-H, Random, 95% CI	Year
**1.1.3 Intensive care unit trials**							
Nobre 2008	8	39	8	40	1.6%	1.03 [0.34, 3.09]	2008
Schroeder 2009	3	14	3	13	0.6%	0.91 [0.15, 5.58]	2009
Hochreiter 2009	15	57	14	53	2.7%	0.99 [0.43, 2.32]	2009
Bouadma 2010	65	307	64	314	13.0%	1.05 [0.71, 1.55]	2010
Layios 2012	56	258	53	251	10.9%	1.04 [0.68, 1.58]	2012
Oliveira 2013	16	49	15	45	2.6%	0.97 [0.41, 2.29]	2013
Annane 2013	7	30	10	28	1.5%	0.55 [0.17, 1.72]	2013
Deliberato 2013	2	42	4	39	0.6%	0.44 [0.08, 2.54]	2013
Shehabi 2014	30	196	26	198	6.1%	1.20 [0.68, 2.11]	2014
Bloos 2016	140	552	149	537	26.9%	0.88 [0.68, 1.16]	2016
De Jong 2016	149	761	196	785	33.5%	0.73 [0.57, 0.93]	2016
**Subtotal (95% CI)**		**2305**		**2303**	**100.0%**	**0.88 [0.76, 1.01]**	
Total events	491		542				
Heterogeneity: Tau² = 0.00; Chi² = 6.21, df = 10 (P = 0.80); I² = 0%							
Test for overall effect: Z = 1.85 (P = 0.06)							
**Total (95% CI)**		**2305**		**2303**	**100.0%**	**0.88 [0.76, 1.01]**	
Total events	491		542				
Heterogeneity: Tau² = 0.00; Chi² = 6.21, df = 10 (P = 0.80); I² = 0%							
Test for overall effect: Z = 1.85 (P = 0.06)							
Test for subgroup differences: Not applicable							

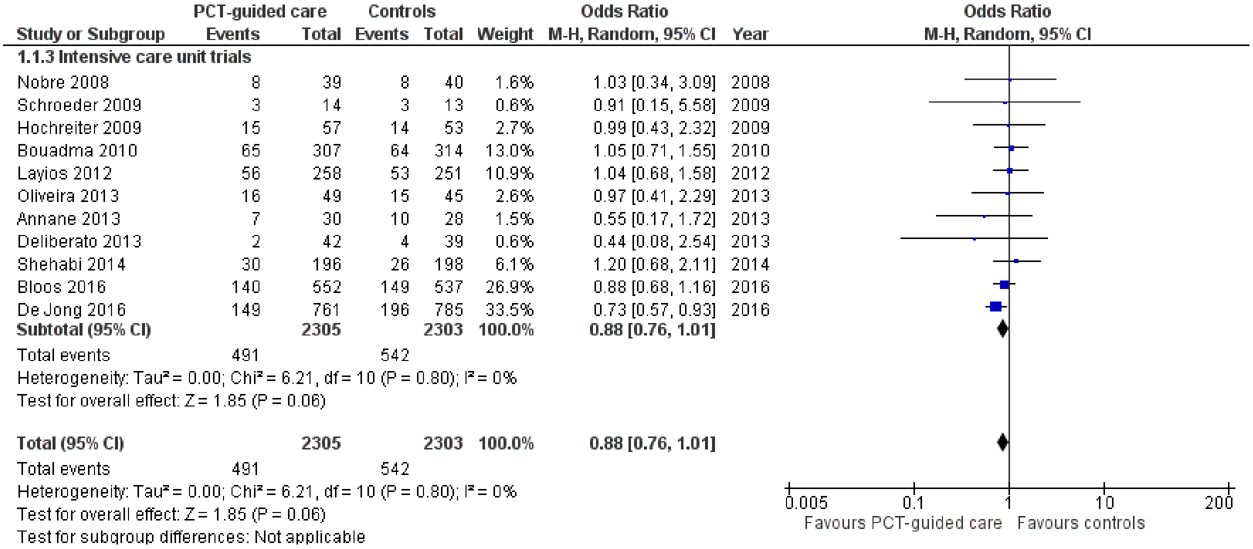

F3 Funnel plots regarding possible publication bias

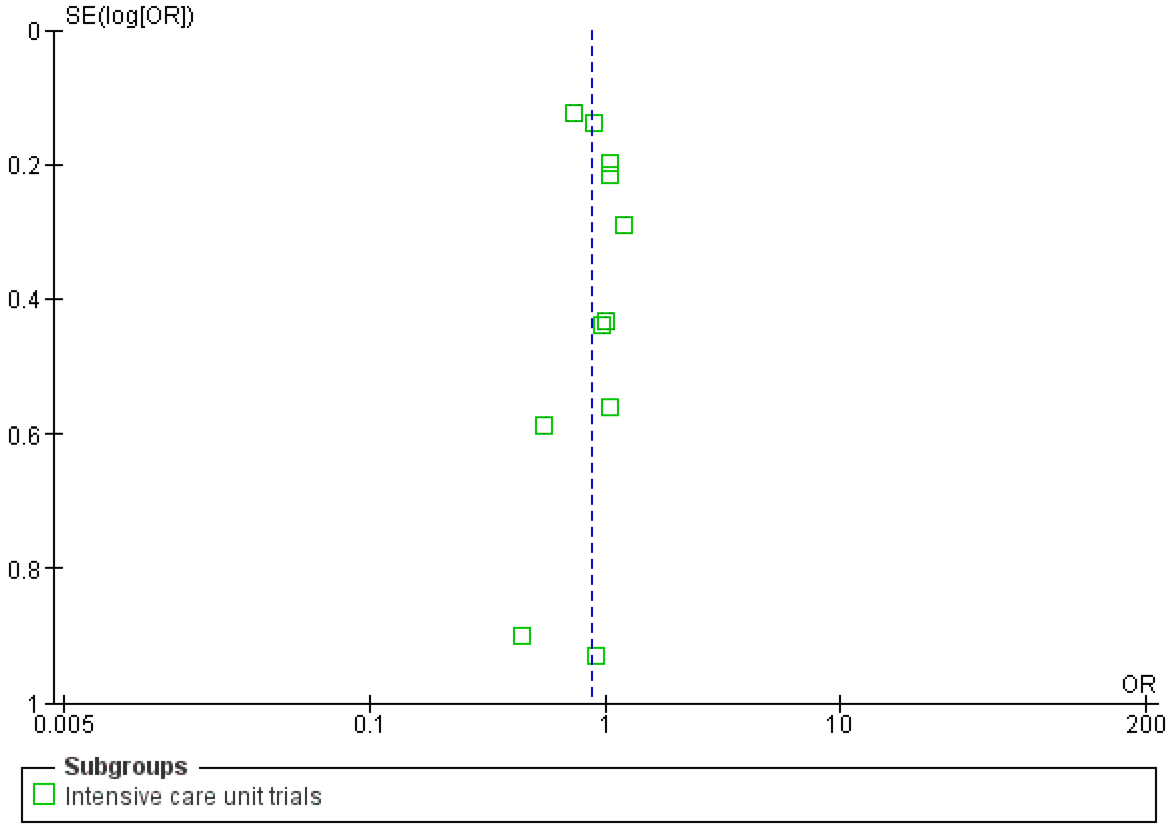

Supplement: Supplementary file 1 — Figure S1. Assessment of risk of bias in included trials. Figure S2. Forrest plot based on aggregate data. Figure S3. Funnel plots regarding possible publication bias. (PDF 57 kb) [file 13054_2018_2125_MOESM1_ESM.pdf]
